# Development of Diagnostic SNP Markers To Monitor Hybridization Between Sika Deer (*Cervus nippon*) and Wapiti (*Cervus elaphus*)

**DOI:** 10.1534/g3.118.200417

**Published:** 2018-05-22

**Authors:** Hengxing Ba, Zhipeng Li, Yifeng Yang, Chunyi Li

**Affiliations:** State Key Laboratory for Molecular Biology of Special Wild Economic Animals, Institute of Special Wild Economic Animals and Plants, Chinese Academy of Agricultural Sciences, No. 4899, Juye Street, Jingyue District, Changchun, Jilin province, 130112, P.R. China

**Keywords:** sika deer, wapiti, hybridization, ddRAD-seq, SNP discovery, STACKS software

## Abstract

Sika deer (*Cervus Nippon*) and wapiti (*Cervus elaphus*) are closely related species and their hybridization can result in significant allele-shift of their gene pool. Additive genetic effects and putative heterotic effects of their hybridization on growth performance could confer considerable economic advantage in deer farming. Here, we used double-digest restriction site-associated DNA sequencing technology (ddRAD-seq) and detected ∼320,000 genome-wide SNPs from 30 captive individuals: 7 sika deer, 6 wapiti and 17 F1 hybrids (reciprocal cross). By screening observed heterozygosity of each SNP across four taxonomic groups, we report for the first time a resource of 2,015 putative diagnostic SNP markers (species-specific SNPs for sika deer and wapiti), which can be used to design tools for assessing or monitoring the degree of hybridization between sika deer and wapiti. These ddRAD-seq data and SNP datasets are also valuable resources for genome-wide studies, including trait discovery for breeders of domestic deer.

Interspecies hybridization is an integral part of evolutionary and speciation processes and is prevalent among plants and lower vertebrate taxa, but in ungulates or other large mammals appears to be rare in nature (Lowe *et al.* 2015; Allendorf *et al.* 2008; Barton 2013, 2001; Mallet 2007). Nevertheless, in the Cervidae hybridization can happen and produce fertile offspring. Some species hybridize naturally in their native range and form hybrid zones, *e.g.*, white-tailed deer (*Odocoileus virginianus*) and mule deer (*Odocoileu Hemionus*) in the USA (Carr *et al*. 1986, Derr *et al.* 1991). Some species which are normally allopatric hybridize when brought into sympatry by man, *e.g.*, red deer (*Cervus elaphus scoticus*) and sika deer (*Cervus nippon nippon*) in Scotland (Goodman *et al.* 1999; Abernethy 1994; Senn and Pemberton 2009; Senn *et al.* 2010). And some species and even genera can be made to hybridize using artificial techniques, *e.g.*, sika (*Cervus nippon nippon*) and wapiti (*Cervus elephus manitobensis*) (Willard *et al.* 1998), sambar deer (*Cervus unicolor*) and red deer (*Cervus elaphus*) (Muir *et al.* 1997), and Pere David’s deer (*Elaphurus davidianus*) and red deer (*Cervus elaphus*) (Tate *et al.* 1995). Therefore, deer species have the potential to be developed as a novel model for understanding mechanisms underlying the phenomenon of fertile hybridization in general.

Deer are farmed for antler velvet or venison in Asia, Europe, and Oceania (Masuko and Souma 2009). In some instances, hybridization may give considerable economic advantages over pure breeds through additive genetic effects and putative heterotic effects on growth performance of farmed deer (Asher *et al.* 1996).

Single nucleotide polymorphic (SNPs) are abundant throughout the genome and mostly biallelic (Lai 2001). Due to the low number of alleles and low degree of homoplasy, SNPs are more likely to be diagnostic than highly polymorphic markers (*e.g.*, microsatellites), providing ideal markers for hybrid analysis. The use of SNPs as probes for determining the degree of hybridization has been applied to a wide variety of taxa (Nussberger *et al.* 2013; Lamer *et al.* 2015; Marques *et al.* 2017). To date, genome-wide SNP discovery in sika deer (Ba *et al.* 2017), white-tailed deer (Seabury *et al.* 2011), and red deer (Brauning *et al.* 2015) has been reported. Very recently, the high-density genetic map of red deer (Johnston *et al.* 2017) (based on 38,000 SNPs) and draft genome of red deer (Bana *et al.* 2018) and reindeer (*R. tarandus*) (Li *et al.* 2017) have also been published. We believe that these deer genetic resources will provide a rich source for further development of genome-wide SNPs within Cervidae.

Here, we employed double-digest restriction-site associated DNA sequencing (ddRAD-seq) technology (Peterson *et al.* 2012) to obtain a dataset of species-specific SNPs that are fixed for a different allele in sika deer and wapiti respectively, meaning that the polymorphism at the SNP was only found between, but not within these two species. These SNPs could be used as candidate diagnostic markers to assess the degree of hybridization between sika deer and wapiti.

## Materials And Methods

### Animals

A total of 30 unrelated animal individuals were selected, including 7 sika deer (*Cervus nippon hortulorum*) (SK), 6 wapiti (*Cervus elaphus xanthopygus*) (WT) and 17 F1 hybrid offspring (produced from reciprocal cross using artificial insemination: sika deer ♀ × wapiti ♂ (9 SW) and wapiti ♀ × sika deer ♂ (8 WS)) (Table 1). The SK and WT were captured from their native region respectively, and transferred to Changbai Mountain Wildlife Experimental Station (Zuojia, Jilin, China). The deer in the station have been served as experimental animals for multiple experiments over the years. Individual animals including sika deer and wapiti were selected based on their pedigree to make sure they were pure and unrelated with each other. The fact that the SK and WT are not parents of the F1 hybrids in this study should be emphasized here.

**Table 1:** Statistics describing different properties of each sequenced individual

Samples	NCBI SRA Accession	# PE clean reads	Base pair (Gbp)	% Overall Mapping	% Unique Mapping with mismatch ≤ 3
SK1	SRR5481378	40,370,837	7.37	82.69%	65.83%
SK2	SRR5481376	42,578,332	7.77	79.97%	63.46%
SK3	SRR5481397	39,682,028	7.24	78.53%	62.56%
SK4	SRR5481381	43,935,174	8.02	81.30%	65.39%
SK5	SRR5481392	46,373,033	8.46	75.62%	59.52%
SK6	SRR5481409	49,799,725	9.09	81.90%	66.51%
SK7	SRR5481408	36,495,356	6.66	86.06%	69.47%
WT1	SRR6765233	39,001,138	7.12	83.31%	61.81%
WT2	SRR6765232	47,783,028	8.72	79.60%	61.54%
WT3	SRR6765231	31,322,025	5.72	79.02%	61.66%
WT4	SRR6765230	33,236,810	6.07	76.17%	58.20%
WT5	SRR6765229	38,863,043	7.09	84.53%	66.16%
WT6	SRR6765228	53,742,306	9.81	25.33%	20.00%
SW1	SRR6765227	37,645,532	6.87	90.07%	76.67%
SW2	SRR6765226	28,954,722	5.29	90.51%	77.32%
SW3	SRR6765235	35,673,032	6.51	90.59%	77.06%
SW4	SRR6765234	31,673,032	5.78	88.62%	72.22%
SW5	SRR6765246	15,081,434	2.75	88.62%	78.14%
SW6	SRR6765245	31,173,032	5.69	76.20%	60.74%
SW7	SRR6765248	30,612,032	5.59	79.96%	63.85%
SW8	SRR6765247	17,850,889	3.26	90.91%	78.26%
SW9	SRR6765242	27,642,132	5.05	78.31%	62.21%
WS1	SRR6765241	37,601,938	6.86	27.17%	21.70%
WS2	SRR6765244	29,632,232	5.41	88.13%	72.20%
WS3	SRR6765243	45,010,955	8.22	78.02%	62.65%
WS4	SRR6765240	34,622,232	6.32	77.29%	61.21%
WS5	SRR6765239	42,912,764	7.83	79.45%	63.16%
WS6	SRR6765236	34,623,732	6.32	76.67%	61.06%
WS7	SRR6765237	56,646,144	10.34	73.79%	57.82%
WS8	SRR6765238	31,225,732	5.70	78.73%	62.46%

### DNA extraction

Whole blood samples were extracted from the jugular vein using EDTA vacuum tubes and were stored at -20° until DNA extraction. Genomic DNA was purified from the collected blood samples using the Blood DNA kit (Qiagen). Each DNA sample was subjected to 0.7% gel electrophoresis for detecting the presence of high-molecular weight DNA and then stored at -80° until ddRAD-Seq library construction.

### Pilot study to identify restriction enzymes

We selected two restriction enzymes for the study based on cutting efficiency from the four enzymes having 6 base recognition sites (*i.e.*, *Pst*I, *NsiI*, *Eco*RI and *Sac*I) and two with 4 base recognition sites (*i.e.*, *Mse*I and *MluCI*). Six digest reactions were carried out based on the protocols by manufacturers: *Mse*I + Buffer 3.1, *Eco*RI + Cutsmart Buffer, *Pst*I + Buffer 3.1, *NsiI* + Buffer 3.1, *Sac*I + Cutsmart Buffer and *MluCI* + Cutsmart Buffer. Both *Pst*I and *Mse*I were selected to construct our ddRAD-Seq libraries because both could be activated in the same buffer and achieve nearly complete digestion (Figure 2A).

### ddRAD-SEQ library preparation and sequencing

The double digest reactions were carried out in a volume of 25 μl containing ∼0.8 μg of genomic DNA, 5U of *Pst*I and *Mse*I, and 1× Buffer 3.1 (NEB). The reaction mixture was incubated at 37° for 2 hr and 65° for 30 min. Amplification and sequencing adapters with a unique barcode (6 bp or 8 bp) were ligated onto the digested DNA. Each sample was then amplified via PCR in a 50 μl reaction volume, containing 70-100 ng of adaptor-ligated DNA fragments and amplified with 22 cycles following the manufacturer’s protocol. Samples were electrophoresed on a 2% agarose gel, and DNA in the 300-450 bp size range (with indices and adaptors) was excised and purified using a Gel Extraction Kit (Qiagen). Each sample library was pooled in equal amounts and quantified using an Agilent 2100 bio analyzer (Agilent Technologies) and then paired-end 101 bp sequencing was performed using the Illumina HiSeq4000 platform at BGI (Shenzhen, China).

### Raw data processing

Low quality reads were filtered or corrected by using the process_radtags program (-c -q –r) in STACKs v2.0 (Catchen *et al.* 2011; Catchen *et al.* 2013), based on the three following rules: 1) remove reads with an uncalled base; 2) discard reads with low quality average scores within the sliding window (15% of the read length) that drop below a raw phred score of 10; 3) rescue ddRAD digestion sites within a certain allowance. Quality of clean reads was checked using FastQC (Andrews 2010).

### ddRAD-SEQ data analysis and SNP inference

**Figure 1:**
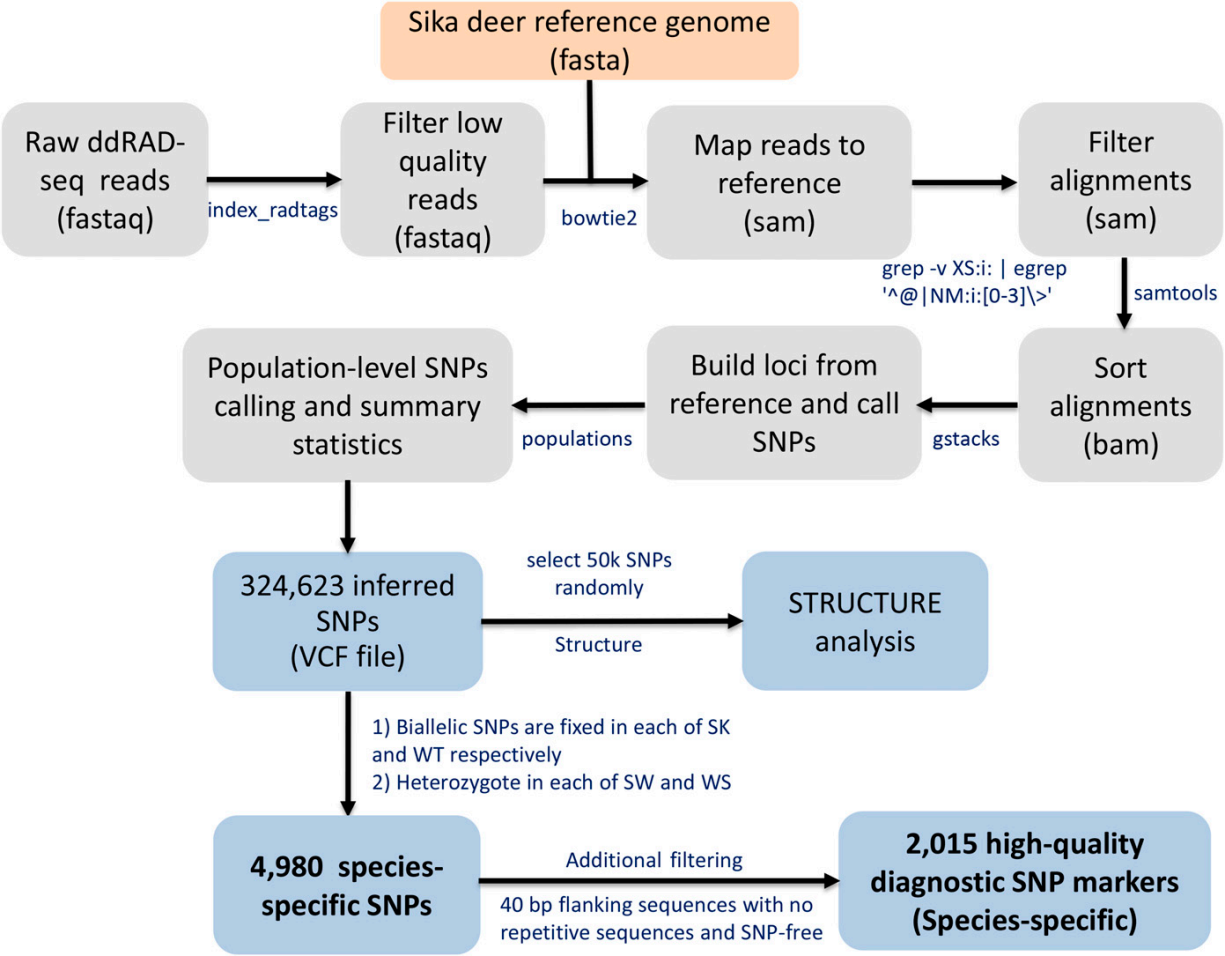
Schematic pipeline of the ddRAD-seq data processing procedures.

The SNP inference process used in this study was an expanded version of a recently published STACKs protocol (Rochette and Catchen 2017), and is illustrated in Figure 1. The filtered paired-end reads from the SK, WT, WS and SW groups were mapped to sika deer reference genome in end-to-end mode using Bowtie2 (Langmead and Salzberg 2012) (disallowing gaps and suppressing unpaired and discordant alignments). The size of this assembled genome is 2.73 Gbp, and its detailed statistics metrics were reported by our previous study (Ba *et al.* 2017). To exclude potential mapping errors, we used the grep -v XS:i: | egrep ’^@|NM:i:[0-3]\>’ command to obtain unique mapping alignments with mismatch ≤3, followed by SAMtools v1.3 (Li 2011) to convert and sort SAM to BAM files.

The BAM files for the all individuals were used as the input of gstacks program in STACKs v2.0 with the marukihigh model (Maruki and Lynch 2017), a type of maximum-likelihood method that is appropriate for high-coverage sequencing data (average 15x per ddRAD locus in this study), and minimum required quality above 20 to call SNP alleles. The populations program (-p 4 -r 0.85–min_maf 0.1–lnl_lim -10.0–merge_sites–renz mseI) in STACKs was subsequently used to produce a VCF file that contains SNP information found in at least 85% of individuals across the four groups and with minor allele frequency ≥0.1. It is notable that we did not specify a maximum H_obs_ required to filter out a nucleotide site at a locus by using the–max_obs_het parameter in the populations program.

### Development of high-quality diagnostic SNP markers

We selected species-specific SNPs as diagnostic SNP markers to meet two filtration criteria: 1) biallelic SNPs are fixed in each individual of the SK and WT groups respectively and 2) all corresponding homologous sites in each individual of the SW and WS groups are heterozygous (observed heterozygosity of all sites are 1). In order to design genotyping assays in the future, sufficient 40 bp flanking sequences with no repetitive sequences and no SNPs within them were also obtained by retrieving the sika deer reference genome.

### Availability of supporting data

The VCF file including SNP information is available from Figshare:10.6084/m9.figshare.5918464. The various genetic parameter statistics for each taxonomic group are available from Figshare: 10.6084/m9.figshare.5918467. The diagnostic SNP markers are available from Table S1. Code used to simulate and produce ddRAD-seq restriction fragments on the genome according to fragment size is available from Figshare: https://doi.org/10.6084/m9.figshare.5942554.

The sika deer ddRAD-seq data can be retrieved from NCBI SRA under BioProject accession number PRJNA383774 (SRA accession number SRR5481378, SRR5481376, SRR5481397, SRR5481381, SRR5481392, SRR5481409 and SRR5481408). The ddRAD-seq data of the wapiti and F1 hybrids were stored under BioProject accession number PRJNA432874. Supplemental material available at Figshare: https://doi.org/10.6084/m9.figshare.6291740.

## Results and Discussion

### ddRAD sequencing and SNP discovery

After performing quality control, we obtained a total of 1.11 billion clean paired-end reads (202 Gb), ranging from 2.75 to 10.34 Gb for each individual with an average of 6.76 Gb (Table 1). The average percentage of unique mapping with mismatch ≤3 is 63.03. We obtained 2,121,131 candidate ddRAD loci with length of 229 ± 71 bp (average depth of 15x) shared by at least 85% of individuals across the four groups. From these ddRAD loci, we inferred 324,623 biallelic SNPs that had minor allele frequency ≥0.1, corresponding to ∼6.7 heterozygous SNPs per Kb. Finally, we selected a subset of 2,015 species-specific SNPs with 40 bp flanking sequences that are fixed for a different allele in sika deer and wapiti respectively (see Materials and Methods).

### To confirm whether ddRAD-SEQ library construction and sequencing data were unbiased and verifiable

**Figure 2:**
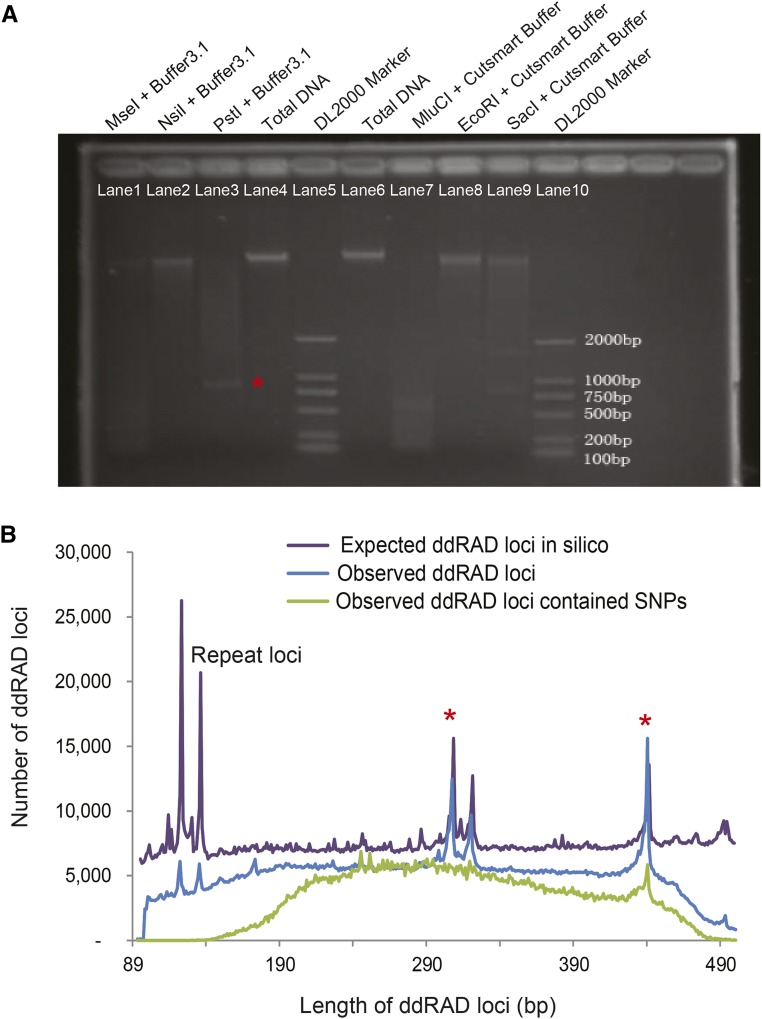
Quality evaluation of ddRAD-Seq library construction. (A) Selection of enzymes for the construction of ddRAD libraries by evaluating cutting efficiency of restrictions enzymes 6 base recognition sites (*i.e.*, *Pst*I, *NsiI*, *Eco*RI and *Sac*I) and 4 base recognition sites (*i.e.*, *Mse*I and *MluCI*). (B) Frequency distribution of restriction fragment length, including the expected 3,049,956 fragment sequences sheared from the reference genome, the observed 2,121,131 loci from ddRAD libraries and those containing SNPs.

In order to examine if the distribution of the observed length of ddRAD loci (2,121,131) produced from the gstacks program perfectly matched with those in the reference genome, we performed a simulation and produced a total of 3,049,956 restriction fragments from the reference genome. Consistent length distribution between the observed and the expected ddRAD loci indicates restriction digestion and sequencing are complete for our ddRAD-seq libraries (Figure 2B). That is, ∼69.5% of restriction sites on the genome are covered by the unique mapping reads (∼63.03%) sequenced from the ddRAD-seq libraries. Note that the short restriction fragments may be the repeat loci, such as ∼122 bp fragments, the number of which is significantly higher than those of the observed ddRAD loci containing SNPs. Highly enriched ∼320 bp and ∼440 bp fragments sheared by both *Pst*I and *Mse*I could correspond to a distinct electrophoresis band in the region between 750 bp and 1000 bp in the *Pst*I lane (not digested by *Mse*I) (Figure 2A red asterisk). Overall, these screening results confirmed that our ddRAD-Seq library construction and sequencing were unbiased and verifiable.

### Confirmation of taxonomic status of groups

**Figure 3:**
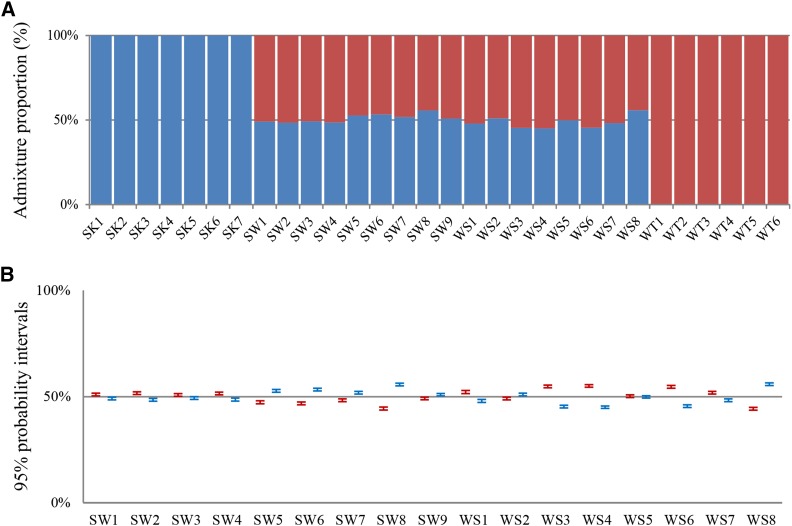
Estimated admixture proportions and cluster membership. Plot of admixture proportion for all individuals (A) and their 95% credible intervals (CIs) for the F1 hybrids (B) for each individuals for K = 2 (number of assumed populations). A dataset of randomly selected 50,000 SNP markers was used for this analysis.

The diagnostic power of SNP markers could be strongly reduced if any of the samples was unrepresentative. STRUCTURE analysis was performed to confirm that each sequenced individual was assigned to its respective population based on its SNP genotypes by using Structure v2.3.4 (Pritchard *et al.* 2000), with three replicate runs and 1 million steps after a burn-in period of 300,000 and K = 2. In order to reduce the linkage probability, a subset of 50,000 SNPs (324,623 SNPs in total) drawn from one random SNP per independent ddRAD locus was analyzed to estimate cluster membership and admixture proportions across the 30 individuals. The admixture proportions were plotted using Excel2010. The results showed the F1 hybrids acquired ∼50% of the genome derived from either of the SK and WT groups respectively (Figure 3A). Based on the estimation of credible intervals (CIs), the admixture proportion of all SK and WT individuals fell within their 95% CIs (0.999 to 1.0), indicating these individuals are pure, and introgression among them did not occur. However, the 95% CIs of almost all F1 hybrids (except for WS5) fell in the region outside the 50% (Figure 3B), which further supports the fact that the SK and WT are not parents of the F1 hybrids in this study. Although the 95% CIs fell in the region outside the 50% for the F1s hybrids, it would not affect development of diagnostic SNP markers to monitor hybridization between sika deer and wapiti.

Theoretically, all biallelic SNPs should be shared among these groups and the private alleles (the alleles that only appear in one of the four groups in the study) should not be detected if sampling number were large enough. We observed only a small subset of 2,086 (0.64%) SNPs that belonged to private alleles calculated by the populations program in STACKs, including 1,386, 352, 308 and 40 SNPs in the SK, WT, SW and WS groups respectively. More convincingly, the private alleles have lower allele frequencies (average MAF = 0.19). These results confirmed that our samples are suitable for the development of diagnostic SNP markers.

**Figure 4:**
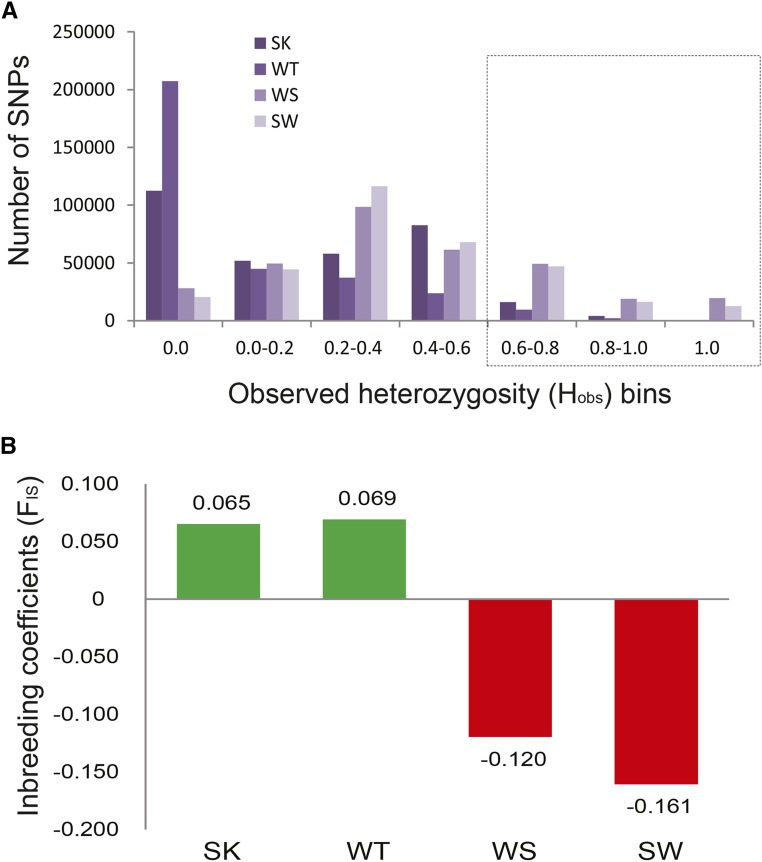
Population genetic parameters for putative SNP loci. (A) Frequency of SNPs across the observed heterozygosity (H_obs_) bins for the four groups. The number of SNPs with H_obs_ ≥ 0.6 was lower in the sika deer and wapiti than in the F1 hybrids. (B) The average coefficients of inbreeding (F_IS_) across all SNPs in pure sika and wapiti were positive while in the F1 hybrids they were negative due to an excess of heterozygotes.

Observed heterozygosity (H_obs_), expected heterozygosity (H_exp_) and inbreeding coefficients (F_IS_) for each SNP within each separate group were calculated by the populations program in STACKs. It is widely known that an observed excess of heterozygotes can be caused by hybridization. Substantial heterozygous SNPs with H_obs_ > 0.6 were observed in the WS and SW, but not in SK and WT (Figure 4A). In addition, the average F_IS_ of each SNP was less than zero in WS and SW, further supporting that the F1 hybrids included an excess of SNP heterozygotes as expected (Figure 4B).

### Conclusions

The ddRAD-seq data and SNP datasets for the sika deer and wapiti are valuable resources to researchers who carry out genome-wide studies, including trait discovery for breeders of farmed deer. The developed diagnostic SNP markers will be useful for the development of hybridization monitoring tools. However, it is noticeable that these diagnostic SNP markers were only inferred from a small population, and therefore any diagnostic genotyping assay built from this dataset should be first tested with adequate sample sizes.

## References

[bib1] Abernethy K., 1994 The establishment of a hybrid zone between red and sika deer (genus Cervus). Mol. Ecol. 3: 551–562. 10.1111/j.1365-294X.1994.tb00086.x 7834107

[bib2] Allendorf F. W. England P. R. Luikart G. Ritchie P. A. Ryman N., 2008 Genetic effects of harvest on wild animal populations. Trends Ecol. Evol. 23: 327–337. 10.1016/j.tree.2008.02.008 18439706

[bib3] Asher G. W. Berg D. K. Beaumont S. Morrow C. J. Neill K. T. O., 1996 Comparison of seasonal changes in reproductive parameters of adult male European fallow deer (Dama dama dama) and hybrid Mesopotamian X European fallow deer (D d mesopotamica X D d dama). Anim. Reprod. Sci. 45: 201–215. 10.1016/S0378-4320(96)01577-1 9227923

[bib4] Ba H. Jia B. Wang G. Yang Y. Kedem G., 2017 Genome-Wide SNP Discovery and Analysis of Genetic Diversity in Farmed Sika Deer (Cervus nippon) in Northeast China Using Double-Digest Restriction Site-Associated DNA Sequencing. G3 (Bethesda) 7: 3169–3176. 10.1534/g3.117.300082 28751500 PMC5592941

[bib5] Bana N. A. Nyiri A. Nagy J. Frank K. Nagy T., 2018 The red deer Cervus elaphus genome CerEla1.0: sequencing, annotating, genes, and chromosomes. Mol. Genet. Genomics 293: 665–684. 10.1007/s00438-017-1412-3 29294181

[bib6] Barton N. H., 2001 The role of hybridization in evolution. Mol. Ecol. 10: 551–568. 10.1046/j.1365-294x.2001.01216.x 11298968

[bib7] Barton N. H., 2013 Does hybridization influence speciation? J. Evol. Biol. 26: 267–269. 10.1111/jeb.12015 23324003

[bib8] Brauning R. Fisher P. J. Mcculloch A. F. Smithies R. J. Ward J. F., 2015 Utilization of high throughput genome sequencing technology for large scale single nucleotide polymorphism discovery in red deer and Canadian elk. bioRxiv. 10.1101/027318

[bib9] Carr S. M. Ballinger S. W. Derr J. N. Blankenship L. H. Bickham J. W., 1986 Mitochondrial DNA analysis of hybridization between sympatric white-tailed deer and mule deer in west Texas. Proc. Natl. Acad. Sci. USA 83: 9576–9580. 10.1073/pnas.83.24.9576 3467326 PMC387183

[bib10] Catchen J. Hohenlohe P. A. Bassham S. Amores A. Cresko W. A., 2013 Stacks: an analysis tool set for population genomics. Mol. Ecol. 22: 3124–3140. 10.1111/mec.12354 23701397 PMC3936987

[bib11] Catchen J. M. Amores A. Hohenlohe P. Cresko W. Postlethwait J. H., 2011 Stacks: building and genotyping Loci de novo from short-read sequences. G3 (Bethesda) 1: 171–182. 10.1534/g3.111.000240 22384329 PMC3276136

[bib12] Derr J. N. Hale D. W. Ellsworth D. L. Bickham J. W., 1991 Fertility in an F1 male hybrid of white-tailed deer (Odocoileus virginianus) x mule deer (O. hemionus). J. Reprod. Fertil. 93: 111–117. 10.1530/jrf.0.0930111 1920279

[bib13] Goodman S. J. Barton N. H. Swanson G. Abernethy K. Pemberton J. M., 1999 Introgression through rare hybridization: A genetic study of a hybrid zone between red and sika deer (genus Cervus) in Argyll, Scotland. Genetics 152: 355–371. 10224266 10.1093/genetics/152.1.355 PMC1460577

[bib14] Johnston S. E. Huisman J. Ellis P. A. Pemberton J. M., 2017 Sexual dimorphism in recombination landscape in red deer (Cervus elaphus): a role for meiotic drive? bioRxiv. 10.1101/100131 PMC5555489 28667018

[bib15] Lai E., 2001 Application of SNP technologies in medicine: Lessons learned and future challenges. Genome Res. 11: 927–929. 10.1101/gr.192301 11381021

[bib16] Lamer J. T. Ruebush B. C. Arbieva Z. H. McClelland M. A. Epifanio J. M., 2015 Diagnostic SNPs reveal widespread introgressive hybridization between introduced bighead and silver carp in the Mississippi River Basin. Mol. Ecol. 24: 3931–3943. 10.1111/mec.13285 26096550

[bib17] Langmead B. Salzberg S. L., 2012 Fast gapped-read alignment with Bowtie 2. Nat. Methods 9: 357–359. 10.1038/nmeth.1923 22388286 PMC3322381

[bib18] Li H., 2011 A statistical framework for SNP calling, mutation discovery, association mapping and population genetical parameter estimation from sequencing data. Bioinformatics 27: 2987–2993. 10.1093/bioinformatics/btr509 21903627 PMC3198575

[bib19] Li Z. Lin Z. Ba H. Chen L. Yang Y., 2017 Draft genome of the reindeer (Rangifer tarandus). Gigascience 6: 1–5. 10.1093/gigascience/gix102 PMC5726476 29099922

[bib20] Lowe W. H. Muhlfeld C. C. Allendorf F. W., 2015 Spatial sorting promotes the spread of maladaptive hybridization. Trends Ecol. Evol. 30: 456–462. 10.1016/j.tree.2015.05.008 26122483

[bib21] Mallet J., 2007 Hybrid speciation. Nature 446: 279–283. 10.1038/nature05706 17361174

[bib22] Marques J. P. Ferreira M. S. Farelo L. Callahan C. M. Hacklander K., 2017 Mountain hare transcriptome and diagnostic markers as resources to monitor hybridization with European hares. Sci. Data 4: 170178 10.1038/sdata.2017.178 29206218 PMC5716010

[bib23] Maruki T. Lynch M., 2017 Genotype Calling from Population-Genomic Sequencing Data. G3 (Bethesda) 7: 1393–1404. 10.1534/g3.117.039008 28108551 PMC5427492

[bib24] Masuko T. Souma K., 2009 Nutritional Physiology of Wild and Domesticated Japanese Sika Deer, pp. 61–82 in Sika Deer: Biology and Management of Native and Introduced Populations, edited by McCullough D. R. Takatsuki S. Kaji K. Springer Japan, Tokyo 10.1007/978-4-431-09429-6_5

[bib25] Muir P. D. Semiadi G. Asher G. W. Broad T. E. Tate M. L., 1997 Sambar deer (Cervus unicolor) x red deer (C-elaphus) interspecies hybrids. J. Hered. 88: 366–372. 10.1093/oxfordjournals.jhered.a023120 9378911

[bib26] Nussberger B. Greminger M. P. Grossen C. Keller L. F. Wandeler P., 2013 Development of SNP markers identifying European wildcats, domestic cats, and their admixed progeny. Mol. Ecol. Resour. 13: 447–460. 10.1111/1755-0998.12075 23398610

[bib27] Peterson B. K. Weber J. N. Kay E. H. Fisher H. S. Hoekstra H. E., 2012 Double digest RADseq: an inexpensive method for de novo SNP discovery and genotyping in model and non-model species. PLoS One 7: e37135 10.1371/journal.pone.0037135 22675423 PMC3365034

[bib28] Pritchard J. K. Stephens M. Donnelly P., 2000 Inference of population structure using multilocus genotype data. Genetics 155: 945–959. 10835412 10.1093/genetics/155.2.945 PMC1461096

[bib29] Rochette N. C. Catchen J. M., 2017 Deriving genotypes from RAD-seq short-read data using Stacks. Nat. Protoc. 12: 2640–2659. 10.1038/nprot.2017.123 29189774

[bib30] Andrews, S., 2010 FastQC: a quality control tool for high throughput sequence data. Available at: http://www.bioinformatics.babraham.ac.uk/projects/fastqc. Accessed: August 3, 2016.

[bib31] Seabury C. M. Bhattarai E. K. Taylor J. F. Viswanathan G. G. Cooper S. M., 2011 Genome-wide polymorphism and comparative analyses in the white-tailed deer (Odocoileus virginianus): a model for conservation genomics. PLoS One 6: e15811 10.1371/journal.pone.0015811 21283515 PMC3023705

[bib32] Senn H. V. Pemberton J. M., 2009 Variable extent of hybridization between invasive sika (Cervus nippon) and native red deer (C. elaphus) in a small geographical area. Mol. Ecol. 18: 862–876. 10.1111/j.1365-294X.2008.04051.x 19175500

[bib33] Senn H. V. Swanson G. M. Goodman S. J. Barton N. H. Pemberton J. M., 2010 Phenotypic correlates of hybridisation between red and sika deer (genus Cervus). J. Anim. Ecol. 79: 414–425. 10.1111/j.1365-2656.2009.01633.x 20002231

[bib34] Tate M. L. Mathias H. C. Fennessy P. F. Dodds K. G. Penty J. M., 1995 A new gene mapping resource: interspecies hybrids between Pere David’s deer (Elaphurus davidianus) and red deer (Cervus elaphus). Genetics 139: 1383–1391. 7768446 10.1093/genetics/139.3.1383 PMC1206464

[bib35] Willard S. T. Flores-Foxworth G. Chapman S. Drew M. L. Hughes D. M., 1998 Hybridization between wapiti (Cervus elephus manitobensis) and sika deer (Cervus nippon): a comparison of two artificial insemination techniques. J. Zoo Wildl. Med. 29: 295–299. 9809601

